# How We Treat Drug-Susceptible Pulmonary Tuberculosis: A Practical Guide for Clinicians

**DOI:** 10.3390/antibiotics12121733

**Published:** 2023-12-14

**Authors:** Niccolò Riccardi, Sara Occhineri, Elisa Vanino, Roberta Maria Antonello, Agostina Pontarelli, Francesca Saluzzo, Tiziana Masini, Giorgio Besozzi, Marina Tadolini, Luigi Codecasa, on behalf of StopTB Italia

**Affiliations:** 1 StopTB Italia ODV, 20159 Milan, Italy; 2 Infectious Diseases Unit, Azienda Ospedaliera Universitaria Pisana, 56124 Pisa, Italy; 3 Infectious Diseases Unit, Santa Maria delle Croci Hospital, AUSL Romagna, 48100 Ravenna, Italy; 4 Unit of Respiratory Infectious Diseases, Cotugno Hospital, Azienda Ospedaliera dei Colli, 80131 Naples, Italy; 5 Emerging Bacterial Pathogens Unit, IRCCS San Raffaele Scientific Institute, Vita-Salute, San Raffaele University, 20132 Milan, Italy; 6 Infectious Disease Unit, IRCCS Azienda Ospedaliero-Universitaria di Bologna, 40138 Bologna, Italy; 7 Department of Medical and Surgical Sciences, Alma Mater Studiorum University of Bologna, 40138 Bologna, Italy; 8 Regional TB Reference Centre, Villa Marelli Institute, ASST Grande Ospedale Metropolitano Niguarda, 20159 Milan, Italy

**Keywords:** tuberculosis, pulmonary TB, drug susceptible, diagnosis, treatment

## Abstract

Tuberculosis (TB) remains one of the leading causes of morbidity and mortality worldwide and pulmonary TB (PTB) is the main variant responsible for fueling transmission of the infection. Effective treatment of drug-susceptible (DS) TB is crucial to avoid the emergence of *Mycobacterium tuberculosis*-resistant strains. In this narrative review, through a fictional suggestive case of DS PTB, we guide the reader in a step-by-step commentary to provide an updated review of current evidence in the management of TB, from diagnosis to post-treatment follow-up. World Health Organization and Centre for Diseases Control (CDC) guidelines for TB, as well as the updated literature, were used to support this manuscript.

## 1. Introduction

Tuberculosis (TB) affects more than 10 million people per year, remaining a major global health threat and one of the leading causes of death from a single infectious pathogen worldwide with 1.30 million deaths in 2022 [1,2].

Affecting people for more than 40.000 years, TB poses never-ending issues in prevention and management, both from a medical and social standpoint. Awareness and knowledge of the disease have deeply improved over the years, but the “modern” history of TB began in 1882 when Robert Koch isolated *Mycobacterium tuberculosis* complex (Mtb) for the first time [3,4]. This, together with the intuition of Rudolf Virchow of the multifactorial genesis of the disease, paved the way to a deeper understanding of transmission dynamics as well as optimizing prevention and treatment strategies [3,4]. 

Mtb is the causative agent of TB; it mainly affects the lungs (85% of cases) but can impact any organ [1]. Mtb is transmitted by aerosol and it is estimated that only 10% of infected people will progress to active disease during their lifetime [5,6]. Several epidemiological and clinical features have been investigated as possible factors that may contribute to acquiring TB infection and to progressing to disease once infected [7]. 

According to the available literature, the most at-risk categories include individuals from high-TB-burden countries, recent contacts of pulmonary TB (PTB), immunocompromised people (e.g., people living with human immunodeficiency virus (PLWH), people treated with anti-TNFα, solid organ or bone marrow transplant recipients), people with chronic diseases (e.g., diabetes, chronic renal failure and dialysis, silicosis, chronic obstructive pulmonary disease), children aged below 5 years, health care workers, migrants, people with a previous TB history, pregnant women, people suffering for malnutrition, people who inject drugs, indigent people and prisoners [1,8,9,10]. 

Timely diagnosis through epidemiological, clinical and radiological suggestive findings is crucial to pursue the World Health Organization (WHO) End TB strategy targets [11,12]. Worldwide, cornerstones of microbiological diagnosis of PTB are still sputum microscopy and culture. However, nucleic acid amplification tests, allowing the rapid identification of mycobacteria genomes and mutations conferring drug resistance (e.g., *rpoB* mutation for rifampicin resistance) have made diagnosis faster and more sensitive and therefore they are currently the primary TB diagnostic test recommended by the WHO [1]. 

The introduction of rifampicin in the 1970s represented a major turning point in drug-susceptible (DS)TB treatment, allowing the use of the first effective shorter course regimen for DS-TB [13,14]. 

According to current international guidelines, first-line treatment for new cases with DS-PTB may last up to 6 months. The standard six-month regimen requires an intensive two-month phase of four drugs (isoniazid (H), rifampicin (R), ethambutol (E) and pyrazinamide (Z)) and a continuation phase of two drugs (H and R) for at least four months [14,15]. 

The length and complexity of treatment represent a challenge to both people living with TB and health care systems, with a reported success rate of 86% [2,14].

In addition to the high number of deaths and the impact on health care-associated costs, TB is also responsible for catastrophic costs for patients and their families [16], clearly summarized in the deprivation of income derived from the partial or total loss of employment [15].

Therefore, prompt diagnosis and effective treatment of TB are crucial to ameliorate the lives of those affected, including their families, to reduce the transmission of the disease and to decrease the likelihood of emergence of resistant strains [17].

In this paper, we provide an example on how to manage DS-PTB along with a comprehensive review of the latest international recommendations.

## 2. Materials and Methods

A typical case of DS-PTB is presented, guiding the reader in a step-by-step commentary supported by an updated review of current evidence in the diagnosis and management of TB, including post-treatment follow-up. The WHO and Centre for Diseases Control (CDC) guidelines, as well as the latest literature, were used to support this didascalic case report and narrative review. This case report is intended to highlight epidemiological, clinical and radiological patterns of pulmonary TB that may be encountered by physicians working in low-TB-burden countries. A web-based literature review was conducted using Pubmed without temporal restriction. Only articles in English and their pertinent references were considered. No informed consent was necessary due to the fictional nature of the case report.

## 3. Case Vignette: Introduction

A 27-year-old man, born in Pakistan and migrated to Italy seven years before, arrived at the emergency department due to hemoptysis and chest pain. His past and recent clinical history was unremarkable and he did not report fever in the previous days. He was living alone and he denied any sick contacts.

At physical examination he appeared underweight (weight 50 kg and height 165 cm: body mass index (BMI) 17.3, resulting in mild thinness); he was breathing normally at rest and at prolonged speech, with peripheral oxygen saturation of 98% in room air; thoracic auscultation showed no pathological lung sounds. Full blood cell count and C-reactive protein were within normal values.

### 3.1. Recommendation in Reference to the Clinical Case

#### Epidemiology and Typical Clinical Manifestation

Clinical findings of PTB include both organ-specific (cough, hemoptysis, exertional dyspnea) and constitutional symptoms such as weight loss, fatigue and low-grade fever [18,19]. Due to the wide differential diagnosis for these symptoms, age and country of origin, together with the risk factor of migration from a high-TB-burden country, resulted in a strong suspicion of TB, or in other words, “tuberculosis until proven otherwise”. This leads to some considerations on epidemiological and personal risk factors.

TB is widespread around the world, with different incidence and prevalence; however, eight countries account for two-thirds of newly diagnosed TB cases each year: India, Indonesia, China, Philippines, Pakistan, Nigeria, Bangladesh and the Democratic Republic of the Congo [2]. A total of thirty countries (all located in Africa and Asia, with the exception of Brazil and Papua Nuova Guinea) have been classified as high-TB-burden countries, and accounted for 87% of incident cases of TB in 2021 [2]. In Europe, Eastern countries have higher incidence rates than Western countries, with Ukraine being the country with the highest TB incidence [2]. Moreover, wars and conflicts increase the risk of reactivation and transmission of TB due to the resulting migration flows, the poor social and hygienic conditions, the impossibility of maintaining prevention programs and the undermining of ongoing treatments, thus also increasing the risk for the emergence of rifampicin-resistant and multidrug-resistant (RR/MDR)-TB strains [20,21].

Alongside the country of origin and its intrinsic risk of exposure, personal factors may modify the course of TB infection. Both socio-economic and medical conditions increase the risk of developing TB disease. Homelessness, sheltered housing conditions, unemployment and migration, especially within the first two years from resettling, are known socio-economic risk factors for TB infection or reactivation [22,23,24].

When individual factors (migration, smoke, intravenous drug use, alcohol abuse, malnutrition, working in health care services, close contact with an active TB case) and underlying medical conditions (HIV infection or other causes of immunosuppression, diabetes, hemodialysis, psychiatric disorders) co-exist, the risk of developing TB disease is further increased [2,25,26,27,28,29,30,31,32].

Keeping all these considerations in mind, TB suspicion based on clinical presentation (i.e., hemoptysis, chest pain, loss of body weight), epidemiology (i.e., country of origin) and risk factors (i.e., migration, although not recent in our case) remains key for a prompt diagnosis and subsequent proper management.

### 3.2. Case Vignette: Diagnostic and Management

**Figure 1:**
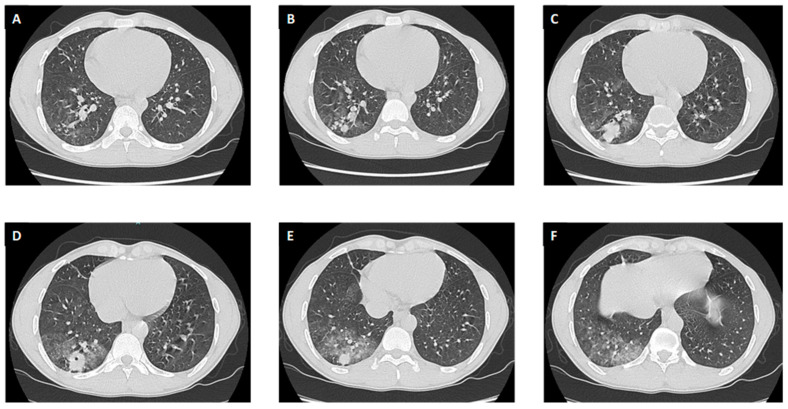
Chest CT scan at presentation (Panel (**A**–**F**)).

In suspicion of active pulmonary bleeding needing vascular embolization, the patient underwent a chest CT scan with iodine contrast that showed a “lung lesion of 23 × 24 mm with tree-in-bud consolidation at the posterior segment of the right inferior lobe with a central cavitation of 5 mm, with arterial and venous phase vascularisation and intralesional haematic infarction” (Figure 1).

Because no signs of active bleeding were detected in the CT scan and considering the stable clinical picture and the housing situation (living alone), the patient was linked to the outpatient TB service.

Due to the high suspicion of TB, two sputum samples in the two following days were collected, both showing negative results at both smear microscopy and rapid molecular test (Xpert MTB/RIF Ultra) and other bacterial and fungal pathogen cultures. 

Afterwards, a bronchoscopy with broncholavage was performed; smear microscopy and molecular test (Xpert MTB/RIF Ultra) showed negative results in bronchoalveolar lavage fluid (BALF). No other bacteria or fungi were detected on BALF culture.

Due to the high suspicion of PTB, while waiting for microbiological results, an empiric antitubercular therapy with HRZE was started.

After twenty days, Mtb was isolated on BALF culture (performed both on solid Lowenstein–Jensen medium and liquid medium using the commercially available gold standard, the culture tube MGIT) and drug susceptibility test (DST) showed full susceptibility to the first-line drugs: HRZE.

In addition, screening for HIV, hepatitis B and C and Strongyloides spp. showed negative results, and glycated hemoglobin was within normal range (29 mmol/mol; normal value < 42 mmol/mol).

Contact tracing was performed on close contacts (friends of the index case that were unemployed at the time of TB diagnosis). The screening with interferon gamma release assays (IGRAs) performed at the time of diagnosis and eight weeks after the last close contact with index patient both showed negative results. As the patient lived alone, there were no household close contacts to screen.

#### 3.2.1. Recommendation in Reference to the Clinical Case

#### 3.2.2. Microbiological Findings

The gold standard method for TB bacteriological confirmation remains mycobacterial culture using commercially available liquid media. This is still the most sensitive method for mycobacteria identification, as well as the main test to verify culture conversion and resolution of the disease from the microbiological point of view. Nonetheless, the use of culture as the primary TB diagnostic test is limited in several high-TB-burden countries because of costs, biosafety requirements and the long time required to obtain results (up to 6 weeks for a negative result) [33]. 

Alongside culture, the other conventional diagnostic test for TB diagnosis is the acid-fast bacilli (AFB) smear microscopy. This test still plays a role in TB initial diagnosis, especially in resource-limited settings, but it is an operator-dependent examination with a lower limit of detection (estimated around 1000–10,000 bacilli per ml of sputum) compared to liquid culture (1–10 colony-forming units (CFUs) per ml) and WHO-recommended rapid molecular tests (Xpert MTB/RIF, approximately 114 CFU/mL, and Xpert MTB/RIF Ultra, approximately 15 CFU/mL). Therefore, the use of rapid molecular assays as initial tests to diagnose TB is recommended by the WHO instead of sputum smear microscopy to increase diagnostic accuracy and shorten the time needed for TB diagnosis and allow for the detection of drug resistance [33,34,35].

The rapid and sensitive molecular tests recommended for the initial detection of Mtb complex and drug resistance include Xpert MTB/RIF Ultra and Xpert MTB/RIF (Cepheid, Sunnyvale, CA, USA); Truenat MTB, MTB Plus and MTB-RIF Dx tests (Molbio Diagnostics, Goa, India); and loop-mediated isothermal amplification (TB-LAMP; Eiken Chemical, Tokyo, Japan) [33]. 

These tests may support the rapid diagnosis of TB and drug resistance, but they should be included in an effective and sound diagnostic algorithm based on the known limitations of the tests as well as on the needs and constraints of the diagnostic network within which these tests are performed. Several diagnostic algorithms are proposed by the WHO, including not only the different microbiological methods but also the clinical manifestations, radiological results, epidemiology and patients’ risk factors [33]. 

Culture and AFB smears remain the main tests to monitor response to treatment [33]. Properly collected sputum, induced sputum, gastric aspirate, bronchial aspirate and BALF can be performed for the diagnosis of PTB. Sputum yields a lower sensitivity, especially in cases of disseminated or interstitial lung involvement, peripheral lesions or lesions not communicating with the bronchial system [36]. Induced sputum, obtained after inhalation of nebulized hypertonic saline solution, showed equal yield to BALF, but it is not always feasible to collect; moreover, bronchoscopy is an invasive procedure and the derived BALF dilutes the mycobacterial load [37,38].

In difficult-to-diagnose cases, if the clinical suspicion is high but epidemiological, clinical and radiological findings are not sufficient for a conclusive diagnosis or there is the need to exclude other possible diagnoses (e.g., tumors, autoimmune diseases, other respiratory pathogens), examination of BALF samples is encouraged [34]. In addition, culture isolation allows for drug susceptibility testing and further microbiological tests (e.g., Whole-Genome Sequencing (WGS)). Currently, less invasive and non-sputum-based approaches are being explored to improve acceptability and feasibility of TB diagnostics [39].

#### 3.2.3. Radiological Findings

Chest X-ray or thorax CT scans may guide clinicians to determine the severity and the extension of the disease and to identify the lung area where to perform broncholavage, if necessary [18].

In fact, radiology plays a major role in validating clinical TB suspicion. For PTB, radiological findings may involve only one lung, both lungs, mediastinum, or lymph nodes. Chest X-rays, most of the time, provide enough information to confirm the clinical suspicion. In doubtful cases, a CT scan is required if the epidemiological, clinical and microbiological criteria are not enough to make a diagnosis [40,41,42]. Magnetic resonance imaging (MRI) is routinely employed in the diagnostic work-up of extrapulmonary TB when involving the central nervous system or the bones or in pediatric age [43,44,45,46,47]. There is also growing interest towards Fluorine-18-fluorodeoxyglucose (18F-FDG) positron emission tomography (PET/PET-CT) as a diagnostic or follow-up tool in TB, but nowadays its possible role is seen as controversial mainly because of its poor specificity with regard to other infectious and non-infectious conditions (i.e., aspergilloma, community- or hospital-acquired pneumonia, malignancies, autoimmune disorders), its lack of usefulness during follow-up due to persistent inflammation and its high cost [48,49,50,51].

The most typical findings in lung parenchyma in patients with PTB are cavitary lesions that are more commonly found in the upper lobes. Among other findings, “tree-in-bud” opacities (multiple branching linear structures that are poorly defined and with a patchy distribution), bronchial wall thickening, patchy consolidations, and calcifications are reported [40]. 

“Tree-in bud” opacities are almost pathognomonic findings in PTB, but can also be observed in other medical conditions [40]. Miliary TB presents with typical bilateral widespread millimetric (1–3 mm) hyperdensities and is more common in young children, elderly or immunocompromised patients. Pleuritis and pleural effusion, as well as nodular opacities in proximity to the pleura, can occur alone or in association with lung parenchyma involvement. Complications include bronchial stenosis, bronchesophageal fistula, and aspergilloma [40,52].

As for the mediastinum, hilar enlargement because of lymphadenopathy can occur in this context. Lymph nodes may appear enlarged or colliquative. Sometimes, a central area of necrosis (hypodensity at CT scan) is also reported.

#### 3.2.4. Screening for Co-Infections and Underlying Medical Conditions

During the diagnostic work-up, or as soon as the TB diagnosis is established, screening for co-infections and comorbidities should be promptly carried out to ensure an optimal management. Among co-infections, whenever TB is diagnosed, HIV infection should always be ruled out and vice versa. In 2021, 6.7% of TB incident cases occurred in PLWH, but it is reported that worldwide up to 18% of TB patients are coinfected with HIV, with a different prevalence based on the setting of investigation [2,53,54]. 

The screening should also address hepatitis B virus (HBV), hepatitis C virus (HCV) infection and specific infectious agents based on epidemiology (e.g., *Strongyloides* spp. and *Schistosoma* spp. serology in patients from endemic countries) and individual risk factors (e.g., galactomannan, cryptococcal antigen and cytomegalovirus viremia in immunosuppressed patients) [55,56,57,58]. 

Among comorbidities, diabetes (i.e., determination of glycated hemoglobin), chronic lung conditions and renal and hepatic impairment should be investigated, as well as non-infectious causes of immunosuppression; these conditions may be associated with poor prognosis and pose relevant issues in the management of TB (e.g., dose adjustment for antitubercular medications, including considering drug–drug interactions mainly in HIV coinfected patients under antiretroviral therapy [59,60,61]. 

In addition, especially when a BALF sample is collected from an immunocompromised patient or a patient with underlying chronic medical conditions (e.g., diabetes), this should be tested for other pathogens, including fungal and opportunistic agents [62,63,64,65].

#### 3.2.5. Outpatient Management

The clinical presentation of our patient highlights the absence of critical clinical conditions and comorbidities; young age, stable vital signs and laboratory tests within normal values, and good housing for spatial isolation, without the presence of immune-compromised people in the households, allow for safe outpatient management and follow-up. Moreover, the chances of infection transmission to household contacts are higher before treatment initiation; in fact, after effective TB treatment is started, the contagiousness drastically decreases. The patient was isolated at home, thus reducing the risk of TB transmission. Treatment and care through outpatient management represents a solid option to improve access to treatment without disrupting patients’ lives [15,66]. 

In addition, screening of close contacts should be performed in agreement with the local public health department. IGRA testing usually performed at baseline and 8–10 weeks from the last contact with the index case is generally considered the screening strategy of choice. In individuals without BCG vaccination, TST is a valuable alternative.

### 3.3. Case Vignette: Treatment

Based on his body weight, the patient was started with daily HRZ as a fixed-dose combination (FDC) tablet (HRZ 50/120/300 mg, six tablets per day in order to reach 300 mg per day of H, 720 mg per day of R and 1800 mg per day of Z) 2 h before breakfast and ethambutol (400 mg tablet, three tabs/day, after breakfast), corresponding to a total of nine tablets administered every day.

As soon as diagnosis was made and treatment initiated, notification of DS-PTB was sent to the local public health and hygiene department in order to ensure contact screening.

When full drug susceptibility of the strain was proven, ethambutol was stopped, thus decreasing tablet burden to six tablets daily. Nutritional consultation was continued and an integrational diet was begun.

After the first two months of treatment, pyrazinamide was stopped, and the continuation phase with HR 300/150 mg in fixed-dose combination, two tablets every day 2 h before breakfast, was initiated for the following four months.

#### 3.3.1. Recommendation in Reference to the Clinical Case

#### 3.3.2. Treatment Indications

As recommended by the WHO, people with DS-PTB should receive a 6-month regimen composed of 2 months of HRZE and 4 months of 4HR (due to the presence of a cavity, prolongation of the continuation phase to 7 months can be considered) [15]. To improve adherence, it is recommended that FDCs are used when available [14,15,67].

People living with HIV should receive the same drug regimen with the same duration of treatment as HIV-negative TB patients [66]. For children aged between 3 months and 16 years with non-severe DS-TB, without suspicion or evidence of MDR/RR-TB and without severe acute malnutrition, a shorter DS-TB regimen composed of 2HRZ(E)/2HR can be given [2,67,68,69,70].

Recently, two shorter regimens have been also approved for use in special cases. For people with DS-pulmonary TB aged 12 years and above, a four-month regimen composed of high doses of rifapentine (P), moxifloxacin (M), H and Z for the first 8 weeks (intensive phase) and a continuation phase of PMH for 9 weeks can be administered. This regimen is recommended also for people with HIV infection if CD4+ cell count is above 100 cell/mmc, but it is not recommended for people with certain forms of extra-pulmonary TB (such as TB meningitis, disseminated TB, osteoarticular TB or abdominal TB) or for pregnant, breastfeeding and postpartum women [66,71]. 

### 3.4. Case Vignette: Treatment Monitoring and Integrated Follow-Up

**Figure 2:**
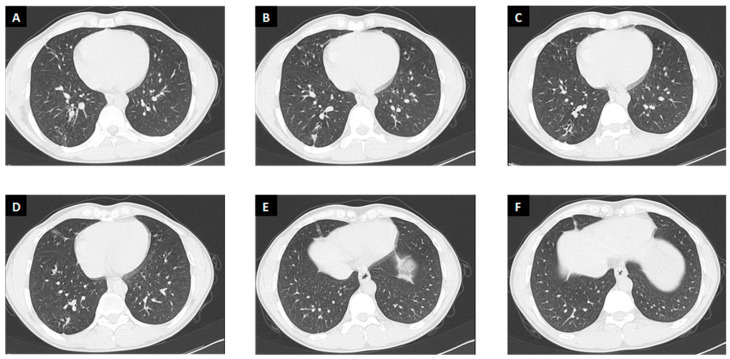
Chest CT scan at the end of treatment (Panel (**A**–**F**)).

After starting therapy, the patient clinically improved with rapid resolution of symptoms and increased weight (BMI 20). Refill of antitubercular drugs was ensured at every outpatient visit (every two weeks for the first month of treatment and then monthly until the end of treatment) to increase adherence to treatment. Blood tests including full blood count and liver function tests were performed monthly without evidence of alterations; glutamic oxaloacetic transaminase (GOT) and glutamic pyruvic transaminase (GPT) were steadily settled between 15 and 35 U/L (normal value 10–50 U/L). At the end of treatment, a thorax CT scan was repeated, identifying a “significant size reduction of the solid lesion excavated at the posterior segment of the right inferior lobe (maximum lesion size of 1 cm) as scars along with traction bronchiectasis” (Figure 2). We did not perform sputum monitoring due to symptoms’ resolution (sputum negative at diagnosis and absence of sputum after treatment initiation) and radiological improvement. Thanks to weekly phone calls with the patient and patient’s relatives and punctual follow-up at outpatient visits, good compliance to treatment was ensured.

#### 3.4.1. Recommendation in Reference to the Clinical Case

#### 3.4.2. Treatment Monitoring and Integrated Follow-Up

All TB cases should be constantly monitored to assess response to treatment. Regular monitoring also facilitates treatment completion and the timely identification and management of adverse drug reactions (discussed later in the manuscript). Patients, their treatment supporters and health workers should be instructed to report the persistence or reappearance of symptoms of TB (including weight loss), symptoms of adverse drug reactions or any treatment interruptions [14,15].

Response to treatment is monitored by clinical progress and through sputum smear microscopy and culture monitoring. For smear-positive DS-PTB patients treated with first-line drugs, sputum smear microscopy may be repeated two weeks after starting therapy and then weekly until negative [14,15].

Sputum-negative patients at baseline need no further sputum monitoring, if treatment adherence is ensured and clinical/radiological improvement is noticed. In case of disease progression, new symptom onset, clinical worsening or poor adherence to treatment, sputum samples should be promptly sent for smear microscopy and culture [14,15].

Constant counseling and health education about the disease are pivotal in TB management [15]. A randomized controlled trial [72] and observational studies revealed a higher treatment success rate and lower loss to follow-up in patients receiving active family support. When feasible, along with constant clinical follow-up, it is essential to provide psychological support to improve adequate compliance in outpatient visits and treatment [15].

Patient weight should be monitored monthly, and drug dosages should be adjusted if weight changes are observed. Nutritional support should be provided to all patients treated for TB to ensure better outcomes. During the treatment and particularly in the first 2 months, weight gain is associated with a reduction in mortality rates [73,74,75]. 

In order to improve adherence, directly observed therapy (DOT), digital adherence technologies (DATs) and patient education programs are fundamental.

Finally, at the end of treatment, according to the WHO definitions, for each patient, treatment outcomes should be reported to local, regional or national public health authorities.

#### 3.4.3. Monitoring of Anti-TB Treatment Side Effects

Regardless of the TB regimen chosen, active clinical and laboratory monitoring is fundamental to ensure rapid identification of any adverse event. Clinicians should know how to manage adverse events to apply timely intervention without compromising treatment success [76,77].

Among the most expected side effects during DS-TB treatment, hepatotoxicity represents the most common and relevant [78,79]. Pyrazinamide is the most frequent culprit [79] of hepatotoxicity. Liver function tests, especially GOT, GPT and total bilirubin serum levels, should be monitored every 15 days to detect early signs of hepatotoxicity and ensure a pre-emptive stop of pyrazinamide if needed [15,78,79].

Patients who suffer from chronic liver disease (e.g., coinfection with viral hepatitis or alcohol abusers) have a higher rate of hepatotoxicity due to pyrazinamide, and thus more frequent monitoring of liver function should be tailored based on comorbidities. Another less common side effect of pyrazinamide is a pruriginous rash that abates after drug suspension. Of note, pyrazinamide can cause cosmetic hyperuricemia, most of the time asymptomatic. In case of renal impairment, pyrazinamide dosage should be adjusted according to renal function [79,80,81].

Rifampicin, one of the cornerstone drugs of DS-TB treatment, may be responsible for various side effects, including cutaneous ones such as flushing on the face and neck, rash or itching, which are usually mild and self-limiting. Hepatitis, and immunologically mediated thrombocytopenia, known since 1970, usually occurs during intermittent treatment with rifampicin and can cause purpura or abnormal bleeding [82,83]. This condition can be reversible with the prompt suspension of the drug [82].

Furthermore, less frequent adverse effects related to rifampicin include shock, shortness of breath, renal failure and hemolytic anemia. In case of the occurrence of any of the latter, immediate suspension of the drug is indicated with no possibility of reintroduction [83]. The incidence of gastrointestinal events (e.g., nausea, loss of appetite, mild abdominal pain, vomiting and diarrhea) due to rifampicin is variable and can be prevented by taking the drug before meals [84,85,86].

Ocular toxicity is a dose-related adverse event associated with ethambutol: a lower dose per Kg (e.g., 15 mg/Kg die) may consent to continue ethambutol without the occurrence of side effects [87,88,89]. Ocular toxicity should be monitored in children treated with ethambutol to prevent retrobulbar neuritis. Educating patients to notice any change in red-green color discrimination can help clinicians to detect early ethambutol toxicity [15]. In case of renal impairment, ethambutol dosage should be adjusted according to renal function [81]. Allergic rash can also occur as a side effect of ethambutol. 

Hepatotoxicity is one of the major isoniazid-related adverse events; the rate of death of isoniazid-induced hepatitis can reach up to 5% among high-risk categories such as people living with HIV, people with alcohol use disorders, people who inject drugs and malnourished people [90]. Peripheral neuropathy is another frequent side effect when isoniazid is taken at conventional dosage in individuals with certain risk factors such as diabetes, alcohol use and malnutrition. This side effect is overcome with pyridoxine supplementation [91,92]. In cases of isoniazid overdose, seizures may appear. Isoniazid can also cause idiosyncratic reactions (e.g., lupus erythematosus and rheumatic-like syndromes) that disappear after discontinuation of the drug [90]. Other uncommon adverse reactions related to isoniazid are dermatitis, fever, angiitis and hemolytic anemia [90]. Treatment with isoniazid is also rarely related to the emergence of secondary manic or psychotic symptoms. Psychiatric manifestations such as irritability, psychomotor agitation, restlessness, euphoric mood and insomnia occur more frequently in patients with latent psychiatric disorders [93,94] and usually appear in the first month of treatment (the latency of psychiatric symptom onset may vary from 1 day to 10 months after the introduction of isoniazid). The occurrence of such side effects requires prompt discontinuation of the drug and psychiatric consultation [93,94].

It is mandatory to evaluate each case of anti-TB-drug-related side effects, and when discontinuation of therapy is deemed unsafe due to the severity of the disease, a regimen based on drugs that do not impact on liver function (in case of previous drug-induced hepatitis) is advised [95].

#### 3.4.4. TB-IRIS and Paradoxical IRIS

TB-associated immune reconstitution inflammatory syndrome (TB-IRIS) is an abnormal and excessive immune response against Mtb. It is a manifestation that can affect people living with HIV after the initiation of antiretroviral therapy (ART), but can also appear in people without HIV infection [96,97]. IRIS is divided into two forms: paradoxical (new manifestation or a worsening of a pre-existing known condition) and unmasking (sudden presentation of a subclinical infection) [96,97]. The clinical presentation includes fever, weight loss, enlargement of lymph nodes and worsening of dyspnea; any organ-specific symptoms can appear if Mtb is localized there (e.g., meningism) [96,97]. Like the IRIS at the initiation of ART, TB-IRIS often happens when the appropriate antitubercular regimen is settled up, generally within three months, but it may appear even after treatment completion. This causes the release of mycobacterial antigens due to their destruction and the activation of the immune system [96,97]. Continuing TB therapy is the appropriate treatment of TB-IRIS with the possible addition of steroids to decrease inflammation [96,97]. 

## 4. Discussion

**Figure 3:**
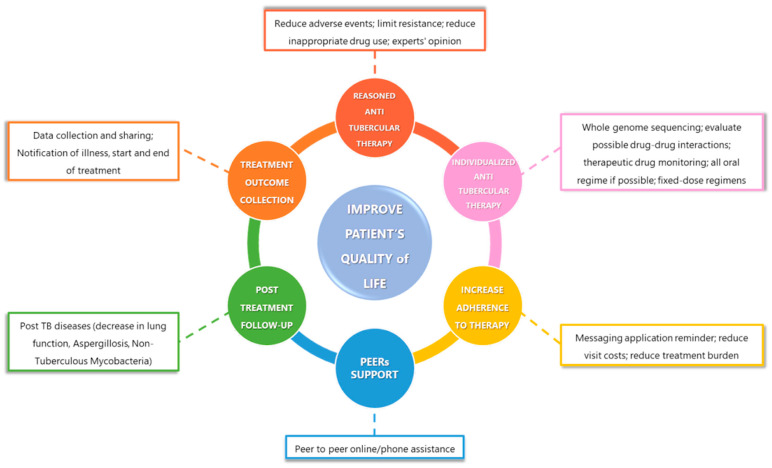
The goals of effective TB care for patients with DS-PTB.

To achieve the ambitious goal of ending the TB pandemic, an early, rapid and accurate diagnosis coupled with effective patient care along treatment pathways is mandatory (Figure 3).

The first challenge of our case report is that rapid microbiological tests (Xpert MTB/RIF Ultra) were negative, both on sputum and broncholavage [98]. Therefore, even if bacteriological confirmation remains a pivotal element of TB diagnostics, it is important to evaluate each case in the appropriate context (taking into account the epidemiology of each patient, close contact of active TB, radiological pattern and clinical presentation) to reach the correct diagnosis. This result also underlines the need for new, less invasive tests for bacteriologically confirming TB diagnosis even in sputum-negative populations or in groups who have difficulties in producing sputum such as children.

As soon as diagnosis is complete, or in cases of clinically diagnosed TB, prompt and effective anti-tubercular treatment based on DST ensures a fast decline in contagiousness, stops clinical and radiological evolution and decreases resistance development [99]. Some considerations about treatment must be underlined: the use of FDCs of anti-TB drugs reduces pill burden, thus increasing treatment adherence. During the long follow-up that follows a diagnosis of TB, it is necessary to evaluate possible drug–drug interactions, and to adjust drug dosage (based on the patient’s weight) at every follow-up visit [15,100,101,102,103]. 

Therapeutic drug monitoring, where available, can be a useful tool for clinicians. A careful examination of drug levels can predict poor response to treatment, drug toxicity, poor drug absorption and drug–drug interactions that can also lead to lower anti-TB drug concentrations [104,105].

While standard treatment for DS-PTB has traditionally been 6 months, new proposed shorter regimens could increase treatment adherence and success. These new therapeutic indications may reduce direct and indirect costs related to disease [14,15,16].

However, the four-month regimen including rifapentine and moxifloxacin is burdened by higher costs (due to rifapentine), a higher pill burden (due to the lack of FDCs) and the lack of widespread availability of rifapentine, which may challenge its implementation [106].

Along with TB treatment, screening for major comorbidities such as HIV and hepatitis virus B and C coinfection, diabetes, malnutrition, chronic pulmonary diseases and geographically specific infections (e.g., Chagas’ disease, strongyloidiasis, schistosomiasis) is necessary to increase patients’ quality of life and TB treatment success [107,108]. Access to drugs is a key issue to ensure successful treatment outcome; governments and national programs need not only to tailor their priority to guarantee drug reimbursement and procurement, but also to support resolutions of people’s stigmatization and unemployment [109,110,111,112,113]. Furthermore, to ensure treatment adherence, it is essential to provide patients with the full drug supply until the next outpatient visit [114].

Another challenge to face in order to reduce TB incidence, mortality and related catastrophic costs is the maintenance of outpatient services during the emergence of pandemics that may disrupt TB services, as happened during the COVID-19 pandemic, or during wars and conflicts [115,116,117].

In our clinical case, outpatient care was appropriate for the patient considering his clinical picture and social conditions; an outpatient model of care should be preferred whenever possible. In fact, studies have shown higher rates of treatment success and lower loss to follow-up when patients were treated with decentralized care versus hospital-based care [15,118]. However, this scenario may not fit all patients, as cases with massive hemoptysis, respiratory failure, laryngeal TB, or severe comorbidities (e.g., severe immunosuppression, children with severe TB, or people who also have advanced HIV) may require hospitalization [118,119].

Furthermore, outpatient care requires properly trained outpatient staff to deliver appropriate care and to support TB patients; clinic staff must also be aware of the early detection and management of adverse drug reactions and should be familiar with social support services. 

The follow-up of DS-PTB patients does not end at treatment completion. In fact, monitoring patients who successfully complete TB treatment allows clinicians to promptly identify any signs of post-tuberculosis lung disease (PTLD) defined as “evidence of chronic respiratory abnormality, with or without symptoms, attributable at least in part to previous pulmonary TB” [115]. The quality of life of these patients is worsened by persistent respiratory symptoms (dyspnea, cough) and reduced capacity for physical exercise. PTLD affects > 50% of patients (>70% for MDR-TB), causing chronic lung functional and structural conditions such as fibrosis, bronchiectasis or pleural thickening [114]. Some patients (about 10% of patients suffering PTLD) lose half of their lung function. These sequelae expose patients to other infections caused by bacteria, viruses, fungi (most of all Aspergillus) and non-tuberculous mycobacteria (NTM) [120].

A low BMI, changes in clinical presentation (such as digital clubbing, raised jugular-venous pressure, peripheral edema) and low peripheral saturation levels must be the focus of attention on patients. A strict monitor of their structural conditions, performed via chest X-ray or CT, and of their functional conditions performed with spirometry, help clinicians to prevent and cure any worsening symptoms promptly and, ultimately, excluding TB relapsing cases [121,122].

Finally, the continuum of care needs to be integrated with peer support (e.g., the possibility for patients to interact by phone or online with former patients), availability of health care staff in case of any new symptoms (e.g., dedicated email and/or dedicated free messaging applications), pro-active contact tracing and screening and outcome notification [2,122].

Treatment support (both psychological and economical) is pivotal to increase people’s compliance with therapy and favorable outcomes. People-centered care based on the patient’s needs and preferences must include psychological and social aspects [14].

## 5. Conclusions

PTB still represents a major global health threat causing millions of deaths worldwide. To end the TB pandemic, every single patient deserves to be treated with the best treatment options available and to be provided with appropriate social support throughout its treatment journey, from access to diagnostic tests to post-treatment follow up.

## Data Availability

Not applicable.
